# A metabolomic signature of decelerated physiological aging in human plasma

**DOI:** 10.1007/s11357-023-00827-0

**Published:** 2023-05-31

**Authors:** Georges E. Janssens, Lotte Grevendonk, Bauke V. Schomakers, Ruben Zapata Perez, Michel van Weeghel, Patrick Schrauwen, Joris Hoeks, Riekelt H. Houtkooper

**Affiliations:** 1 Laboratory Genetic Metabolic Diseases, Amsterdam UMC Location University of Amsterdam, Meibergdreef 9, Amsterdam, The Netherlands; 2 Amsterdam Gastroenterology, Endocrinology and Metabolism, Amsterdam, The Netherlands; 3 Department of Nutrition and Movement Sciences, NUTRIM School of Nutrition and Translational Research in Metabolism, Maastricht University, 6200 MD Maastricht, The Netherlands; 4 TI Food and Nutrition, PO Box 557, 6700 AN Wageningen, The Netherlands; 5 Core Facility Metabolomics, Amsterdam UMC Location University of Amsterdam, Meibergdreef 9, Amsterdam, The Netherlands; 6 Faculty of Health Sciences, UCAM – Universidad Católica de Murcia, 30107 Murcia, Spain; 7 Amsterdam Cardiovascular Sciences, Amsterdam, The Netherlands

**Keywords:** Healthy longevity, Metabolomics, Physiological aging, PhysiAge, Aging clock, TCA cycle, Malate, Citrate, Isocitrate, Aging interventions

## Abstract

The degenerative processes that occur during aging increase the risk of disease and impaired health. Meanwhile, interventions that target aging to promote healthy longevity are gaining interest, both academically and in the public. While nutritional and physical interventions exist, efficacy is often difficult to determine. It is therefore imperative that an aging score measuring the biological aging process is available to the wider public. However, simple, interpret, and accessible biological aging scores are lacking. Here, we developed PhysiAge, a physiological aging score based on five accessible parameters that have influence on or reflect the aging process: (1) average daily step count, (2) blood glucose, (3) systolic blood pressure, (4) sex, and (5) age. Here, we found that compared to calendar age alone, PhysiAge better predicts mortality, as well as established muscle aging markers such as decrease in NAD^+^ levels, increase in oxidative stress, and decline in physical functioning. In order to demonstrate the usefulness of PhysiAge in identifying relevant factors associated with decelerated aging, we calculated PhysiAges for a cohort of aged individuals and obtained mass spectrometry-based blood plasma metabolomic profiles for each individual. Here, we identified a metabolic signature of decelerated aging, which included components of the TCA cycle, including malate, citrate, and isocitrate. Higher abundance of these metabolites was associated with decelerated aging, in line with supplementation studies in model organisms. PhysiAge represents an accessible way for people to track and intervene in their aging trajectories, and identifies a metabolic signature of decelerated aging in human blood plasma, which can be further studied for its causal involvement in human aging.

**Supplementary Information:**

The online version contains supplementary material available at 10.1007/s11357-023-00827-0.

## Introduction

The degenerative processes that occur during aging increase the risk of disease and impaired health. Meanwhile, interventions that target aging to promote healthy longevity are gaining repute and interest, academically and in public and private sectors [1, 2]. While nutritional and physical interventions exist, their efficacy is often difficult to determine. Accommodating this, a wide variety of aging scores and aging clocks exist, including DNA methylation-based, transcriptomics-based, proteomics-based, and metabolomics-based molecular aging clocks [3–6]. While these “omics” clocks are plentiful and allow the determination of a biological, rather than chronological age, they suffer from limited accessibility to the general public. Other clocks that are not based on molecular biology tools, such as those based on blood biochemistry markers [7] or movement patterns from wearable devices [8] are more amenable to public adoption. Nonetheless, arguably most of these forms of clocks suffer from a lack of interpretability. Namely, most of the current aging clocks do not easily offer insights for how an individual could make changes to their lives to alter the clock’s aging predictions.

Since aging clocks can reveal which individuals are biologically younger, aging clocks can also be used to identify factors associated with decelerated aging, with the potential for these factors to be causally involved. For example, our previous work, building a machine-learning based aging clock using accelerometer-based movement patterns, identified nutritional components associated with decelerated aging [8]. These included fiber, magnesium, and vitamin E, as well as the alpha-blocker drug doxazosin, which indeed increased healthspan and lifespan in the nematode *Caenorhabditis elegans* [8]. Furthermore, a number of other factors associated with decelerated aging may have causal involvement. For example, higher diet quality has been related to decelerated aging using epigenetic clocks [9], and diets such as the Mediterranean diet are associated with reduced mortality rates and longer telomere lengths [10]. Likewise, mood stabilizers detected in the blood of patients with bipolar disorders have been related to decelerated aging using an epigenetic clock [11], and this class of drugs (including lithium carbonate, sodium valproate, and carbamazepine) can extend lifespan and/or healthspan in model organisms [12–14]. Taken together, these studies suggest that factors associated with decelerated aging can represent more than just associations and hold promise for causal involvement and treatments.

Metabolism plays a key role in aging [15], and identifying metabolites that associate with decelerated aging represents one of the lowest hanging fruits for developing aging interventions that can be made widely available to the general public. Although not as potent as drugs, metabolites offer the possibility to be administered as supplements, not requiring the same lengthy approval necessary for drug treatments. Therefore, applying aging clocks to aged individuals, and assessing the metabolomes of these individuals, offers an unprecedented opportunity to find factors for future follow-up in human trials. Altogether, it is of high relevance to identify metabolic factors that potentially serve as prophylactic treatments for the general population to promote and maintain health during aging, in addition to developing accessible and interpretable biological aging clocks.

To address these multiple critical elements remaining in the field of gerontology, specifically (1) creation of accessible aging clocks and (2) identification of metabolites associated with decelerated aging, we developed a simple and interpretable aging clock, termed PhysiAge, and applied this to aged individuals for which we attained blood plasma metabolomics data. PhysiAge is built on easy-access data—including average steps-per-day, blood-glucose levels, blood pressure, sex, and age—and provides insights to its user, demonstrating how altered activity (e.g., influencing step count) or diet (e.g., indirectly influencing blood glucose) can influence the rate of physiological aging. We further evaluated our physiological aging score in a small cohort well characterized in terms of its physiological health levels [16]. Finally, we performed metabolomics on blood plasma from these same individuals to identify circulating factors associated with decelerated aging, which is assessed comparing an individual’s PhysiAge to their chronological age. We found a metabolomics signature for decelerated aging that included an increase in TCA cycle constituents, several of which have been linked to healthspan improvements in simple model organisms. These metabolites hence constitute strong candidates to be further studied for their causal involvement in human aging.

## Results

### A strategy for physiological age estimation

**Fig. 1:**
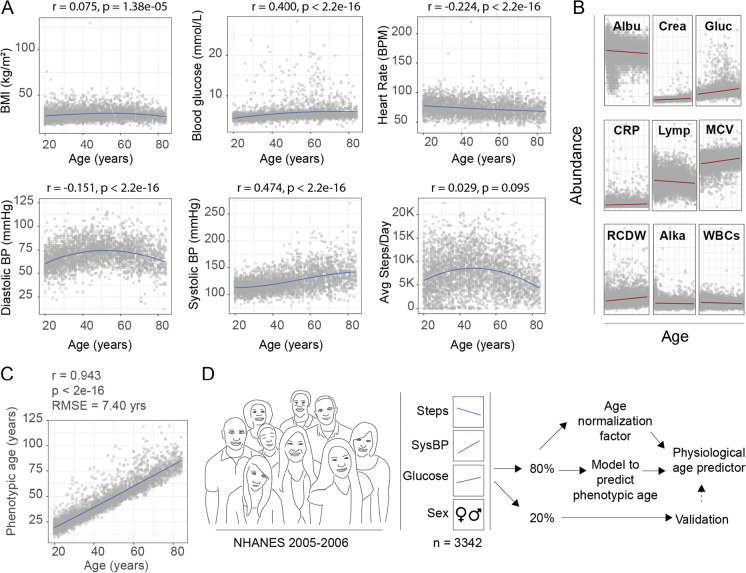
Physiological and phenotypic parameters and a strategy for physiological age prediction. **A** Comparisons of various physiological parameters to age in the NHANES 2005–2006 study year assessed by Spearman correlations. Body mass index (BMI) (rho = 0.075, *p* = 1.38e-05), blood glucose (rho = 0.400, *p* < 2.2e-16), resting heart rate (BPM = beats per minute, rho =  − 0.224, *p* < 2.2e-16), diastolic blood pressure (diastolic BP) (rho =  − 0.151, *p* < 2.2e-16), systolic blood pressure (systolic BP) (rho = 0.474, *p* < 2.2e-16), and average steps per day (rho = 0.029, *p* = 0.095) all showed significant changes with age. **B** The nine blood biochemistry markers used to calculate phenotypic age [7] include albumin (albu), creatinine (crea), blood glucose (Gluc), C-reactive protein (CRP), lymphocyte percent (Lymp), mean cell volume (MCV), red cell distribution width (RCDW), alkaline phosphatase (Alka), white blood cell count (WBCs), in addition to calendar age. **C** Correlation of individual’s phenotypic age with their age (Spearman’s rho = 0.943, *p* < 2.2e-16, RMSE = 7.40 years) in the NHANES 2005–2006 study population used. **D** Strategy for model building to assess physiological age. NHANES 2005–2006 study year possessing data for average steps per say (steps), systolic blood pressure (SysBP), blood glucose (Glucose), and sex (*n* = 3342) were split into training (80%) and testing (20%) datasets and were used to the predict phenotypic age of an individual. An age normalization factor was applied. Final results were validated in the testing dataset (20%). All associations are assessed using Spearman’s correlation where *r* is Spearman’s rho with corresponding *p* value (*n* = 3342)

In order to identify potential physiological markers that change with age and which could be used in a physiological aging score, we turned to the NHANES database, a publicly available resource that contains demographic data, blood biochemistry markers, surveys, mortality records, and more, for a wide range of different ages of individuals (up to age 84, whereby those older are hard coded as 85 +). We specifically focused on the 2005–2006 study years, which were unique in that participants wore activity trackers that quantified their step-count throughout the day for a period of up to 7 days. In an initial exploration of the data, we selected eight parameters for visualization which have all been described to change with age or health, including body mass index (BMI) [17], blood glucose [18], resting heart rate (RHR) [19], systolic and diastolic blood pressure [20], and average steps per day [21], since we considered these as relatively easy for individuals to track themselves (Fig. 1A). Upon visual inspection, we noted that some of these parameters possessed dynamic relationships to age, such as diastolic blood pressure, while others had milder associations, such as with BMI, and some possessed strong and near-linear relations, such as systolic blood pressure (Fig. 1A).

To form the most relevant physiological aging score reflecting an individual’s biological aging rate, we next calculated for each individual their “phenotypic age” based on the work of Levine and colleagues [7], which determines an aging score using nine blood markers that change with age; albumin (Albu), creatinine (Crea), blood glucose (Gluc), C-reactive protein (CRP), lymphocyte percent (Lymp), mean cell volume (MCV), red cell distribution width (RCDW), alkaline phosphatase (Alka), and white blood cell count (WBCs) (Fig. 1B) in relation to an individual’s calendar age [7]. This phenotypic age is especially useful as it is associated with mortality and reflects a better approximation of biological age than calendar age alone [7]. Furthermore, blood biochemistry markers required to calculate phenotypic age are available for most of the NHANES participants of the 2005–2006 study period. Indeed, we found phenotypic age to correlate well to calendar age in our study population (Spearman’s rho = 0.943, *p* < 2.2e-16, *R* mean squared error (RMSE) 7.40 years) (Fig. 1C). Therefore, analogous to how certain DNA methylation clocks have been trained using PhenoAge rather than calendar age for improved assessment of biological aging [7], we aimed to build our own aging clock predicting PhenoAge, based on simple and accessible parameters.

Following this, we next outlined a general strategy to calculate a physiological aging score using easily trackable parameters that could allow for a simple interpretation. To achieve this, we selected physiological parameters that could linearly be considered “good” or “bad,” in the context of the aging process. This included systolic blood pressure and blood sugar, which nearly linearly increased with age, and are associated with age-related diseases including dementia, heart disease, and metabolic syndromes [22–24]. We also included average daily step count since a high daily step count is associated with a lower risk of all-cause mortality in the elderly [21]. Parameters that were less obviously directionally linked to aging, such as diastolic blood pressure, or that were not necessarily directly related to “more” being clearly “better” or “worse”, such as BMI or resting heart rate, were omitted from our model, to preserve our goal of attaining a simple, interpretable, final model. Furthermore, we also planned to apply a normalization factor based on an individual’s calendar age, to adjust for systematic over or under estimations of age and provide values relative to other individuals in the population (Fig. 1D). After assessing data for quality and completeness, 3342 individuals possessed data for all relevant parameters. We planned to use 80% of the NHANES public data for model building and to generate a normalization factor, and reserved 20% for validation and testing associations with mortality (Fig. 1D).

### PhysiAge relates to mortality and offers aging insights

**Fig. 2:**
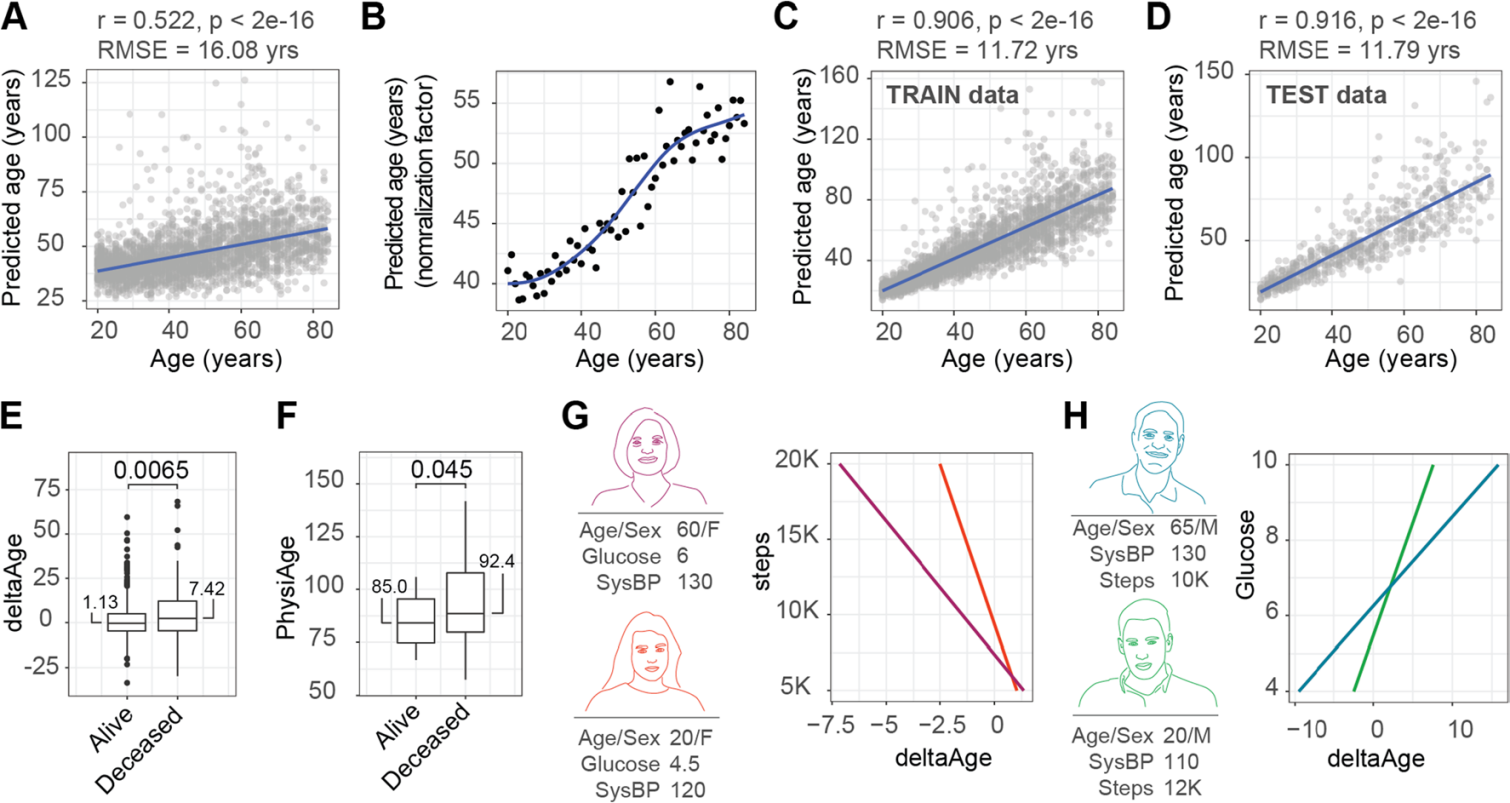
PhysiAge, mortality, and aging insights. **A** Association of model prediction to age (Spearman correlation, rho = 0.522, *p* < 2e-16, RMSE = 16.08, *n* = 2673). **B** Comparison of median predicted age to calendar age, used to generate normalization factor (blue fit). **C** Association of model prediction to age after implementation of normalization factor on the training data (Spearman correlation, rho = 0.906, *p* < 2e-16, RMSE = 11.72, *n* = 2673). **D** Association of model prediction to age after implementation of normalization factor on the testing data (Spearman correlation, rho = 0.916, *p* < 2e-16, RMSE = 11.79, *n* = 669). **E** Comparison of deltaAge of individuals presumed alive (*n* = 597, median deltaAge 1.13 years) and found deceased (*n* = 72, median deltaAge 7.42 years) in the NHANES mortality analysis performed in 2015. Demonstrates that PhysiAge distinguishes individuals more likely to die across the whole population (Students’ *t* test, two-tailed, *p* = 0.0065). **F** Comparison of age prediction of individuals hardcoded as being 85 + , either found alive (*n* = 18, median PhysiAge 85.0 years) or deceased (*n* = 63, median PhysiAge 92.4 years) in the NHANES mortality analysis performed in 2015. Demonstrates that PhysiAge distinguishes individuals more likely to die in the oldest old (Students’ *t* test, two-tailed, *p* = 0.045). **G** Depiction of how varying steps per day influence PhysiAge in women of two different ages. **H** Depiction of how different blood glucose levels influence PhysiAge in men of two different ages

We proceeded to build a multiple linear model using our training data (*n* = 2673), and found that all parameters used were significantly associated with phenotypic age and contributed to the model’s prediction (sex *p* = 0.00173, blood glucose *p* < 2e-16, average step count *p* = 9.73e-10, systolic blood pressure *p* < 2e-16). When evaluating the prediction’s results on the training data, we found a Spearman’s correlation of our prediction to calendar age of 0.522 (*p* < 2e-16) and RMSE of 16.08 years (Fig. 2A). Indeed, the predicted ages remained within a narrow range across the population (~ 20–60 years), not representing the age range of the original population (Fig. 2A). Therefore, we generate a normalization factor using an approach we previously developed [8], based on the median age predicted across the population for any given age (Fig. 2B, Supplemental Table 1). For each individual, we divided their predicted age by the normalization factor relevant for their calendar age, and multiplied this result by their calendar age. This aligned our predicted ages with the chronological ages in the population, and produced a final physiological age prediction (termed PhysiAge) with a Spearman’s correlation of 0.906 to calendar age (*p* < 2e-16) and an RMSE of 11.72 years (Fig. 2C).

In order to validate our results independently of the data used to generate the model and normalization, we looked at the ~ 20% remaining data used for testing the model (*n* = 669). Using the model and normalization factors derived from the training data, we found that the test data resulted in a physiological age prediction with a Spearman correlation of 0.916 to calendar age (*p* < 2e-16) and an RMSE of 11.79 years (Fig. 2C).

We next accessed the mortality records of the NHANES participants, which were available in a follow-up evaluation conducted ~ 10 years after the initial 2005–2006 survey was performed. Using the test data, we calculated the difference between our physiological age prediction and calendar age, termed deltaAge. Negative deltaAges indicated age deceleration, while positive deltaAges indicated age acceleration. We found that while individuals still alive in the follow-up time (*n* = 597) had a mean deltaAge of 1.12 years, those found deceased (*n* = 72) had a mean deltaAge of 7.4 years, over 6 years older (Student’s *t* test, two tailed, *p* = 0.0065). To further validate this finding, we looked at individuals that were scored as 85 + year olds in the NHANES survey. These individuals were not used when building the PhysiAge model as their exact age was unknown. Their sex, blood glucose, average steps, and systolic blood pressure were known however, and we used these parameters with a default age of 85 years to predict their PhysiAge scores. Here, we found that those still alive in the follow-up period (*n* = 18) had a mean predicted age of 85.01, while those found deceased (*n* = 63) had a mean predicted age of 92.43, over 7 years older than their peers who remained alive (Student’s *t* test, two tailed, *p* = 0.045). Taken together, we concluded that PhysiAge captures critical elements of the biological aging process, reflected in mortality.

Since PhysiAge is built on markers that are modifiable by lifestyle, especially average steps per day and blood glucose, we next aimed to illustrate how a PhysiAge prediction can provide insights for how a person should modify their lifestyle to affect their physiological age. In a first assessment looking at average steps per day, we defined two representative females, one older, with an age of 60 years, blood glucose level of 6.0 mmol/L and systolic blood pressure of 130 mmHg, and one younger, with an age of 20, blood glucose level of 4.6 mmol/L, and systolic blood pressure of 120 mmHg. Here, we illustrated how average steps per day influenced the PhysiAge score of the individuals (Fig. 2G). For example, the 60-year-old woman having a step count of 5 K would place her PhysiAge at 61.34, while increasing this to 12 K would result in a PhysiAge of 57.38, nearly 4 years younger (Fig. 2G). Meanwhile, the 20-year-old woman could maintain her PhysiAge of 20.09 years with an average step count of 9 K per day (Fig. 2G).

Following this, we also illustrated how the blood glucose range could affect hypothetical individuals, and defined one older man of 65 years, with a systolic blood pressure of 130 mmHg and average daily step count of 10 K, and another younger man of age 20 years, with a systolic blood pressure of 110 mmHg and average daily step count of 12 K (Fig. 2H). Here, we illustrate how maintaining a low blood glucose levels of, e.g., 5.0 can give both the older and younger men a PhysiAge score lower than their actual age, of 59.71 and 19.21 years, respectively (Fig. 2H). Taken together, we illustrate here how PhysiAge can offer insights into how lifestyle choices, such as exercise (influencing average steps per day) and diet (influencing blood glucose levels), can be harnessed to influence the rate of physiological aging.

### Further statistical validation of the PhysiAge model

**Fig. 3:**
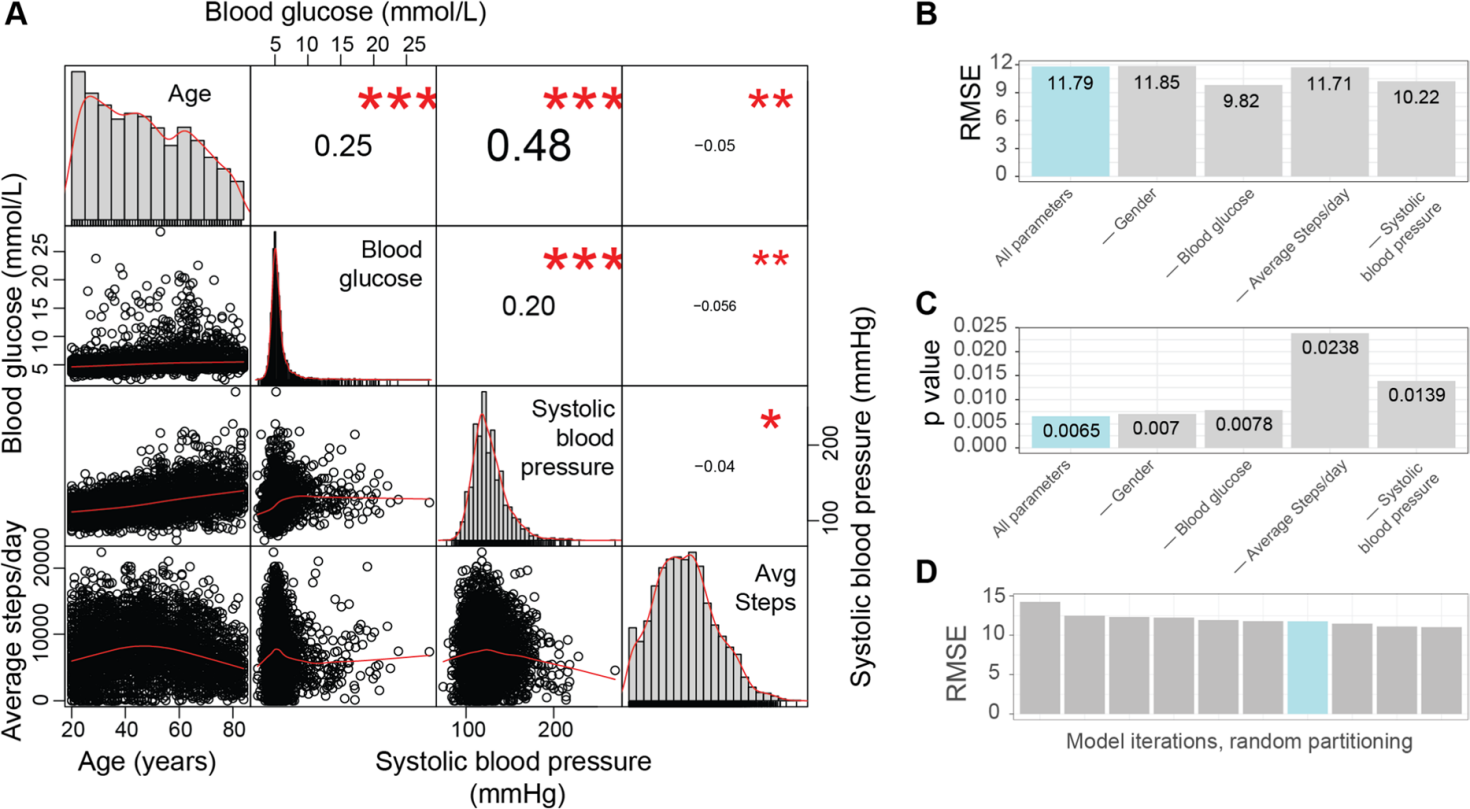
Further statistical validation of the PhysiAge model. **A** Cross-correlation of parameters used in PhysiAge, including Age, blood glucose, systolic blood pressure, and average (avg) daily steps. Shows significant cross-correlation of parameters, though in general each parameter correlates more strongly with age than any other alone (Pearson correlation, * *p* < 0.05, ** *p* < 0.01, *** *p* < 0.001). **B** *R* mean squared errors (RMSEs) of various versions of the PhysiAge model, either including all parameters (“All parameters”) or omitting a single parameter (listed with a “—”). RMSE is based on the holdout dataset reserved for testing of the model. Corresponds to Fig. 2D. **C** Significance level (*p* values) of various versions of the PhysiAge model, either including all parameters (“All parameters”) or omitting a single parameter (listed with a “—”), when comparing the alive and deceased individuals from the NHANES follow-up evaluation period. Results are based on the holdout dataset reserved for testing of the model. Corresponds to Fig. 2E. **D** RMSE of the PhysiAge model using different randomly generated datasplits for training and testing datasets. Demonstrates that the final model is not over-fit due to a specific data split

Next, we aimed to further validate the robustness of our final PhysiAge model. Specifically, we aimed to further evaluate the importance of each parameter in PhysiAge. First, we performed cross-correlations of each continuous parameter to one another. We found Pearson correlations of systolic blood pressure to be greatest to age (0.48), compared to the correlation with blood glucose (0.20) or average daily steps (− 0.04, Fig. 3A). Furthermore, blood glucose correlated strongest to age as well (0.25), compared to blood pressure (0.20) or average daily steps (− 0.04, Fig. 3A). This suggested that both blood glucose and blood pressure contributed independently to PhysiAge. Average daily steps possessed a higher correlation to blood glucose, though this was similar to its correlation to age (− 0.05, Fig. 3A). Taken together, this further suggested that all parameters contributed to the physiological age-predictive capability of our model. In order to further evaluate this however, we proceeded to re-build multiple versions of the PhysiAge model leaving out each of the four parameters once (gender, blood glucose, systolic blood pressure, average daily steps), and compared this to the final PhysiAge model that possessed all five parameters. As a metric to evaluate model performance, we looked at RMSE of the models when assessing the testing dataset (Fig. 3B), and how significantly the models could differentiate between live and deceased individuals in the follow-up period of analysis (Fig. 3C). With this, we used the same approach that we originally used when validating our model (Fig. 2D for RMSE and Fig. 2E for mortality significance, respectively). Interestingly, while maintaining all five parameters produced an RMSE of 11.79 years as expected, removing parameters could improve the RMSE down to 9.82 years, specifically when removing blood glucose (Fig. 3B). However, this improvement in RMSE came at the cost of a poorer separation of live and deceased individuals in the follow-up period, where our original PhysiAge model, which included all five parameters, outperformed all others (Fig. 3C). This suggested that a lower RMSE may not necessarily be better for any particular biological aging clock, if it removes actually biologically relevant information that is present in the model’s error. This result also confirmed that all parameters significantly contribute to PhysiAge’s biological aging score. Finally, to more robustly assess our initial RMSE of 11.79, we generated different versions of the PhysiAge model using different random data splits. We found that our RMSE (11.79) was similar to other versions of the model generated by different data splits (maximum RMSE of 14.27, minimum RMSE of 11.03, median RMSE of 11.87, Fig. 3D). Taken together, these findings all demonstrate that the PhysiAge model benefits from all parameters in its ability to determine physiological age and the PhysiAge model is not over-fit to any particular random split of the training and testing data.

### PhysiAge better predicts age-related decline in muscle health

**Fig. 4:**
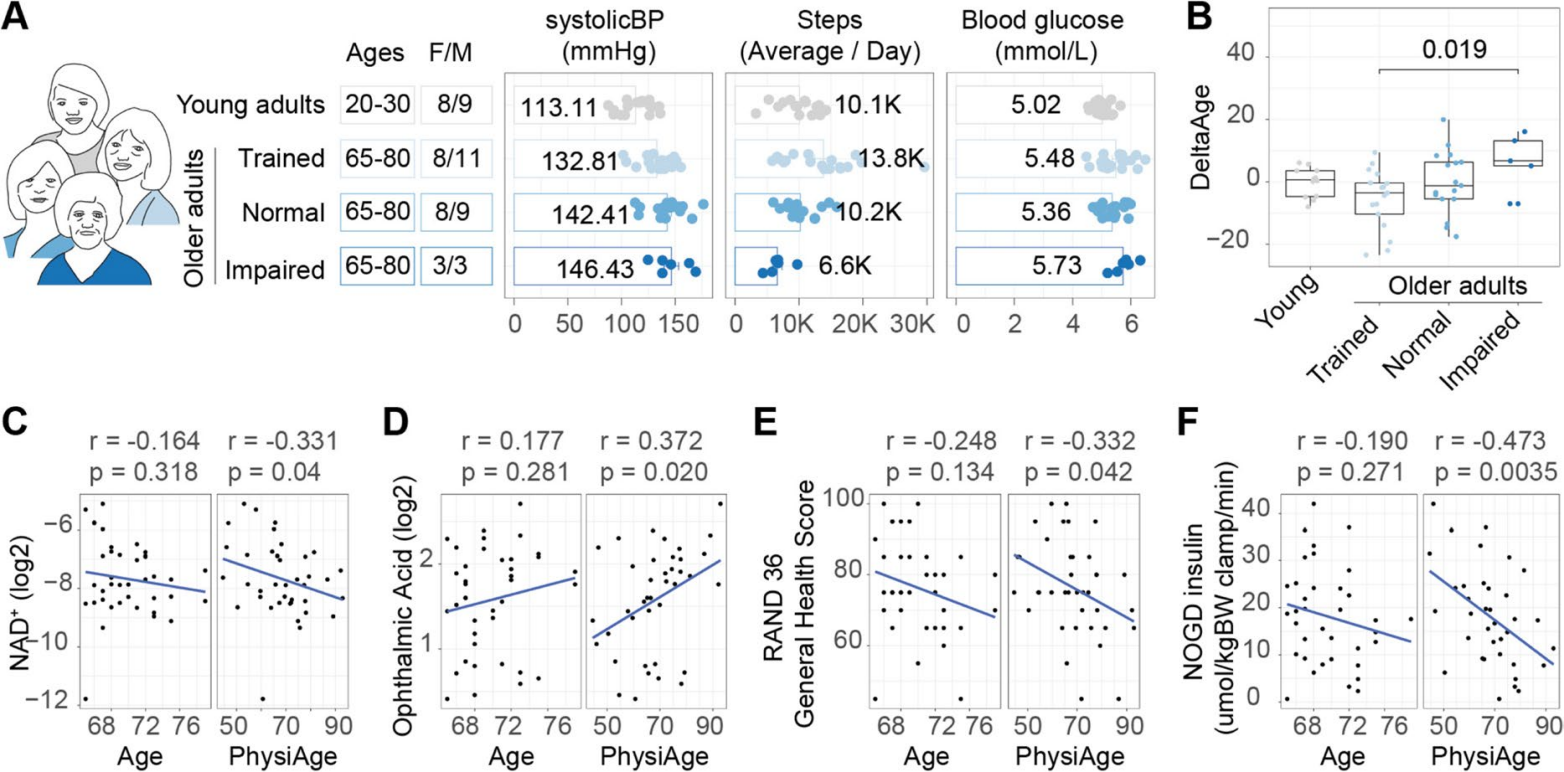
PhysiAge and NAD^+^, oxidative stress, and physiological functioning. **A** The cohort (*n* = 59) used in this study consisted of women and men, either young (20–30) or older (65–80), whereby the old were segmented into different health states, either considered to be athletically trained, normal, or impaired in their aging state. The cohort possessed diverse levels of the parameters used to measure PhysiAge, including systolic blood pressure, average daily step count, and blood glucose. **B** Calculation of deltaAge (PhysiAge minus calendar age) for the individuals in the cohort, grouped by age/health state. A significant difference between the deltaAge of the trained and impaired elderly was detected (Kruskal-Wallis test, *p* = 0.019; Young *n* = 17, older adults trained *n* = 19, normal *n* = 17, impaired *n* = 6). **C** Correlation of NAD^+^ to either age (left panel) or PhysiAge (right panel). **D** Correlation of OPA to either age (left panel) or PhysiAge (right panel). **E** Correlation of RAND 36 to either age (left panel) or PhysiAge (right panel). **F** Correlation of NOGD to either age (left panel) or PhysiAge (right panel). All associations are assessed using Spearman’s correlation with rho and corresponding *p* value (*n* = 59)

In order to further evaluate the relevance of our PhysiAge score to physiological aging, we turned to a small and highly characterized human aging cohort we previously worked with and extensively phenotyped [16, 25]. The cohort consisted of women and men who were categorized as either young (ages 20–30) or older (ages 65–80) individuals, whereby the older adults were further segmented to reflect their health levels, based on their physical training status and (impaired) physical abilities. The groups included either exercise trained older adults (self-assessed as performing at least three structured exercise sessions of at least 1 h duration per week), normal older adults (performing no more than one structured exercise session per week), or physically impaired older adults (empirically assessed using a Short Physical Performance Battery (SPPB) test and classified with a score ≤ 9) [16]. From this cohort, 59 individuals possessed complete data on the parameters necessary to calculate PhysiAge (young *n* = 17, older adults, trained *n* = 19, normal *n* = 17, impaired *n* = 6), and they showed a broad range of values for systolic blood pressure, average daily step count, and fasting blood glucose levels (Fig. 4A).

As an initial characterization, we calculated for each individual in each group their PhysiAge, which showed good correlation to calendar age (Spearman’s rho = 0.805, *p* = 1.027e-12, RMSE = 9.27 years). We subsequently calculated their deltaAge, where we found the young possessed a median deltaAge of 0.642, trained elderly a median deltaAge of − 3.559, normal elderly a deltaAge of − 1.204, and impaired elderly a deltaAge of 6.718. This was in part to be expected from the a priori knowledge that trained older adults inherently possessed greater step counts than the impaired older adults (Fig. 3A). However, our score allowed for a quantification of the extent to which the trained older adults were biologically younger than the impaired older adults. Of note, the trained elderly had a significantly lower deltaAge compared to the impaired elderly (Kruskal–Wallis test, *p* = 0.019, Fig. 4B), constituting over 10 years of age difference between the two groups. Taken together, this indicates that PhysiAge accurately predicted the aging state reflecting the different health groups of the aged individuals.

We next aimed to evaluate how PhysiAge related to multiple critical aging parameters previously measured in this cohort. First, we turned to our existing muscle metabolomics dataset [25], in order to specifically evaluate how the metabolite nicotinamide adenine dinucleotide (NAD^+^), a marker of mitochondrial and metabolic health [26], and ophthalmic acid (OPA), a marker of oxidative stress, both relevant for aging, related to PhysiAge in the older adults. While NAD^+^ showed a slight negative correlation with age of − 0.164, we found that physiAge possessed a stronger negative correlation of − 0.331 to NAD^+^ (Spearman correlation, *p* = 0.04) (Fig. 4C). Likewise, while OPA possessed a slight positive correlation with age of 0.177, physiAge possessed a stronger positive correlation of 0.372 (*p* = 0.020) (Fig. 4D). This demonstrates that physiAge’s age predictions reflect the underlying molecular biology influencing the aging process.

We continued to evaluate two more markers that are especially representative of healthy aging and were characterized for this cohort. These included the RAND 36-item survey for self-rated general health, and the rate of non-oxidative glucose disposal (NOGD). The RAND 36 is on a scale of 0 to 100 where higher indicates better self-rated health. The NOGD is a proxy for insulin-stimulated glycogen storage, where higher is also better. Here, we found that the RAND 36 possessed a trend for a negative correlation with age, which was markedly significant when considering PhysiAge (Spearman’s rho =  − 0.332, *p* = 0.042). We also found a strikingly strong negative correlation of NOGD to PhysiAge (Spearman’s rho =  − 0.473, *p* = 0.0035), which was minor when compared to age alone (Spearman’s rho =  − 0.190, *p* = 0.271). Taken together, these findings indicate that physiAge holds better associations with the physiological aging parameters compared to age alone.

### A metabolomics signature of decelerated aging in blood plasma

**Fig. 5:**
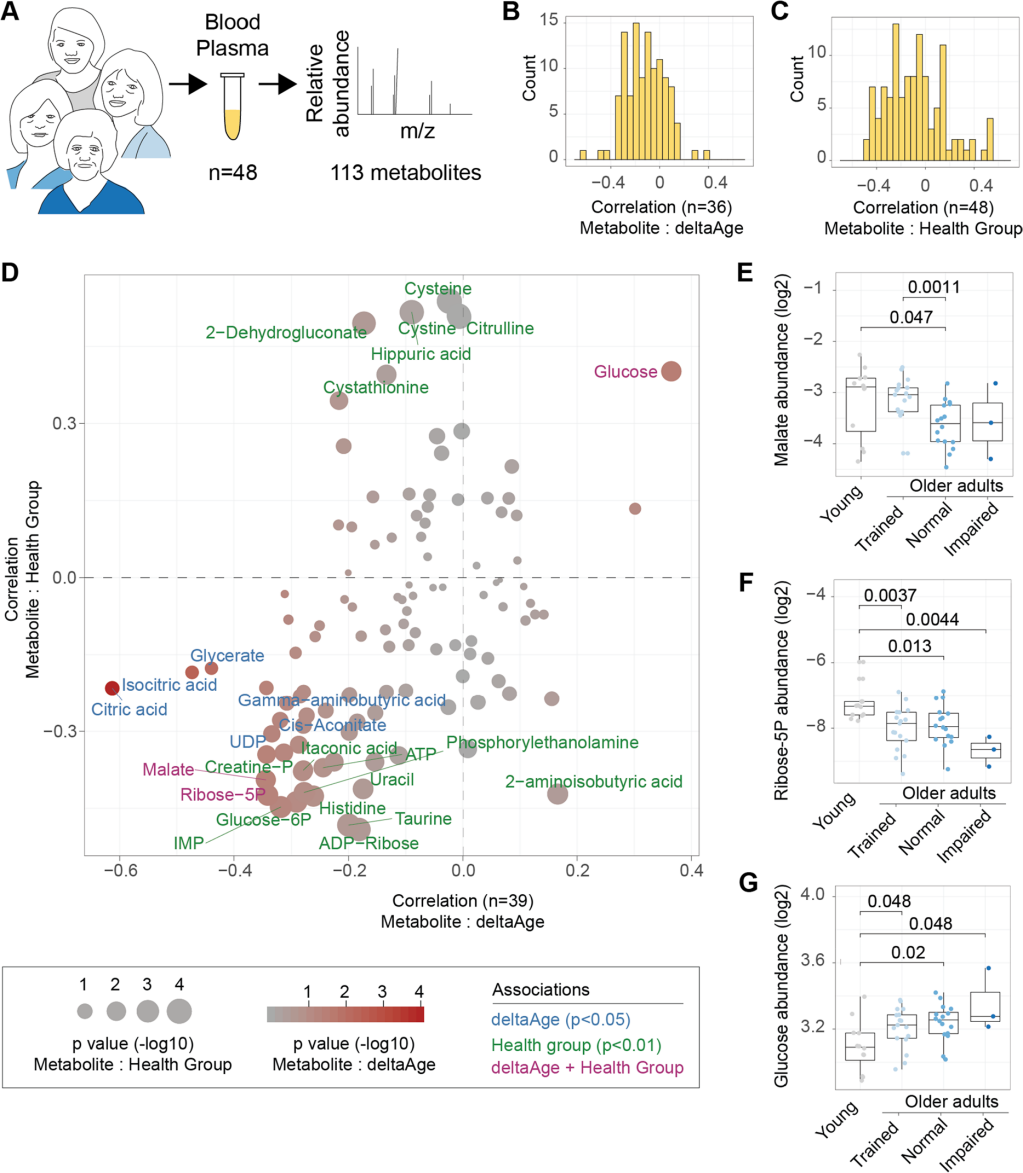
Metabolic signatures of decelerated aging. **A** Ultra-high-performance liquid chromatography high-resolution mass spectrometry (UPLC-HRMS) metabolomics was performed on 48 samples and 113 unique metabolites were annotated. **B** Distributions of the correlations that metabolites have to deltaAge. Spearman correlation using all older adults, *n* = 36. **C** Distribution of the correlations that metabolites have to the four aging/health groups of the cohort. Spearman correlation using all participants, *n* = 48. **D** Scatterplot comparing the metabolite correlation with deltaAge (*x* axis) or the four health groups (*y* axis). Sizes represent –log10 *p* value of the correlation (health group) while color denotes –log10 *p* value of correlation (deltaAge). Metabolites with text written in red were significant in both analyses. **E** Comparison of malate abundance across the four aging/health groups of the cohort. **F** Comparison of ribose 5-phosphate abundance across the four aging/health groups of the cohort. **G** Comparison of glucose abundance across the four aging/health groups of the cohort. For all group comparisons (panels **D**–**F**), the Kruskal–Wallis test is used to compare differences. Young *n* = 12. Older adults trained *n* = 17, normal *n* = 16, impaired *n* = 3

Having observed that PhysiAge could identify metabolic changes representative of accelerated or decelerated aging in muscle, including the decline of NAD^+^ and increase in the oxidative stress marker OPA, we next asked whether we could use PhysiAge to identify metabolomic signatures of decelerated aging in blood plasma. Blood plasma is especially interesting since circulating metabolite levels provide a window into physiological processes and homeostasis in the whole organism. We therefore turned to the same highly characterized independent cohort used to assess PhysiAge (Fig. 3A) [16, 25] and collected plasma samples from these individuals. We performed ultra-high-performance liquid chromatography high-resolution mass spectrometry (UPLC-HRMS) semi-targeted metabolomics on 48 available samples, allowing us to annotate 113 unique and validated metabolites (Fig. 5A, Supplemental Table 2).

Following this, we proceeded to check how each metabolite’s abundance correlated to two different markers of aging health. Firstly, we correlated the metabolite levels to the deltaAge of the aged individuals based on their PhysiAge scores (*n* = 36). This stratified the metabolites across a scale of being more or less related to age acceleration or deceleration (Fig. 5B). Secondly, we correlated the metabolite levels of all individuals to the age/health groups of the cohort (*n* = 48). This served to identify metabolites correlating either positively or negatively with a health trend as defined by the four groups (Fig. 5C). We found that both results were well correlated to one another (Spearman’s rho = 0.479, *p*-value = 7.776e-08).

In order to identify metabolic signatures of decelerated aging and health, we then cross-compared these results to identify metabolites in common between these two analyses, or unique to them. We found nine metabolites associated either positively or negatively to deltaAge (Spearman correlation, *p* < 0.05; Fig. 5D, blue text), and twenty metabolites associated to the four health groups (Spearman correlation, *p* < 0.01; Fig. 5D, green text). Interestingly, using these cutoffs, three metabolites were in common between the two analyses, including malate and ribose 5-phosphate, which were associated to age deceleration and better health states, and glucose, which was associated with age acceleration and poorer health states (Fig. 5D, purple text). Malate showed significantly lower levels in the normal older adults compared to young (*p* = 0.047), which was not seen in the trained older adults compared to young (Fig. 5E). Ribose 5-phosphate was lowest in the impaired older adults compared to young (*p* = 0.0044), though also low in the normal (*p* = 0.013) and trained (*p* = 0.0037) older adults compared to young (Fig. 5F). Finally, glucose showed a step-wise and significant increase in all of the older adults compared to young, likely due to its role in our PhysiAge model prediction (Fig. 5G).

**Fig. 6:**
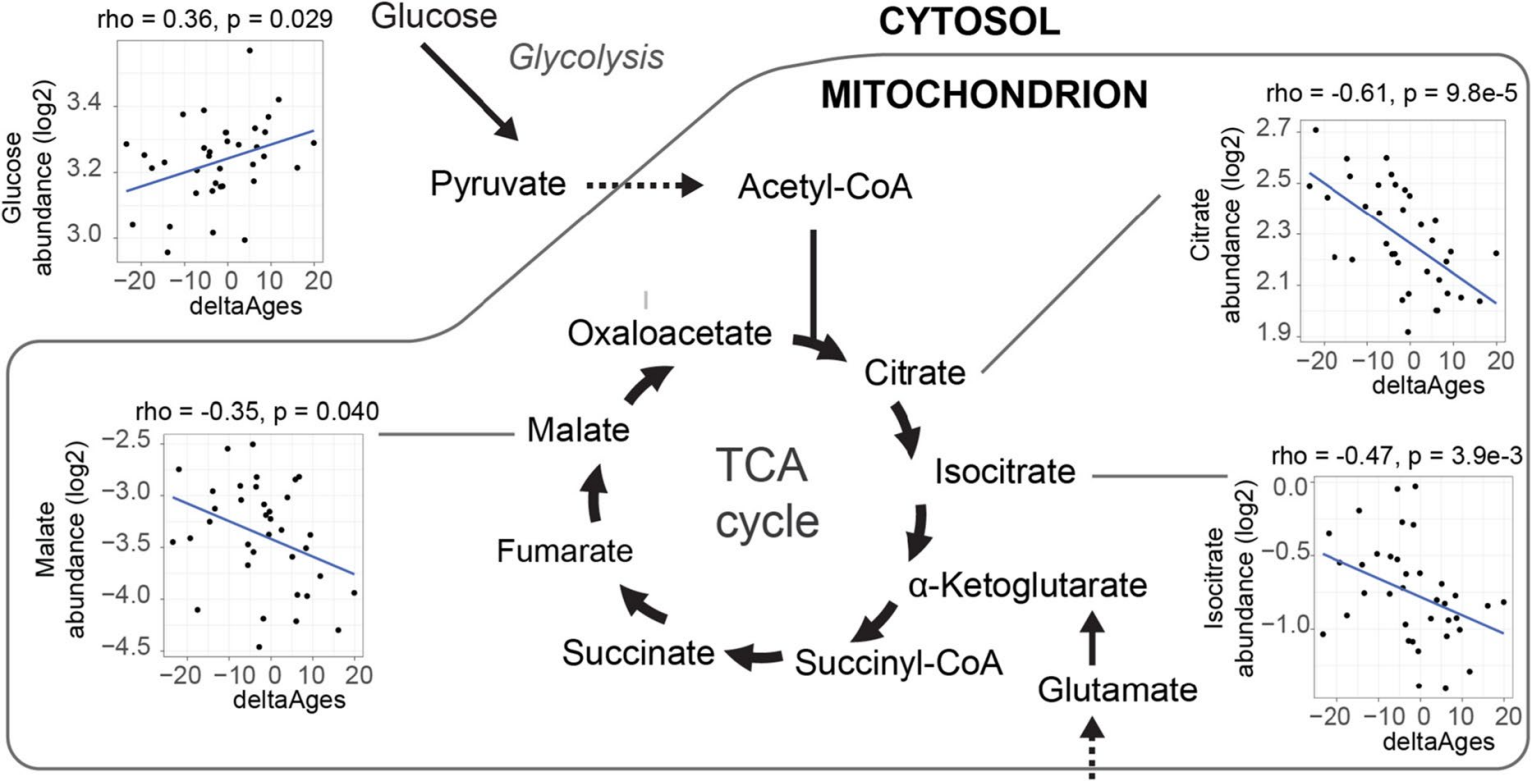
Tricarboxylic acid (TCA) cycle and decelerated aging. Multiple metabolites of the tricarboxylic acid (TCA) cycle were associated with decelerated aging, including citrate (Spearman’s rho =  − 0.61, *p* = 9.8e-5), isocitrate (Spearman’s rho =  − 0.47, *p* = 3.9e-3), and malate (Spearman’s rho =  − 0.35, *p* = 0.040). Glucose, which through glycolysis forms pyruvate which is converted to acetyl-CoA and feeds the TCA cycle, is positively correlated with accelerated aging (Spearman’s rho = 0.36, *p* = 0.029)

Malate, which was significantly negatively correlated to deltaAge (Spearman’s rho =  − 0.35, *p* = 0.040), is an intermediate of the tricarboxylic acid (TCA) cycle, and we noted that two other metabolites of the TCA cycle were associated with decelerated aging when comparing deltaAge to metabolite abundance, namely citrate (Spearman’s rho =  − 0.61, *p* = 9.8e-5) and isocitrate (Spearman’s rho =  − 0.47, *p* = 3.9e-3) (Fig. 6). These showed negative correlations to deltaAge, specifically implying that higher levels were associated with younger (decelerated) physiological age, while lower levels were associated with older (accelerated) physiological age. Taken together, our work here implicates a metabolic signature of decelerated and healthy aging, involving ribose 5-phosphate and implicating the TCA cycle.

**Fig. 7:**
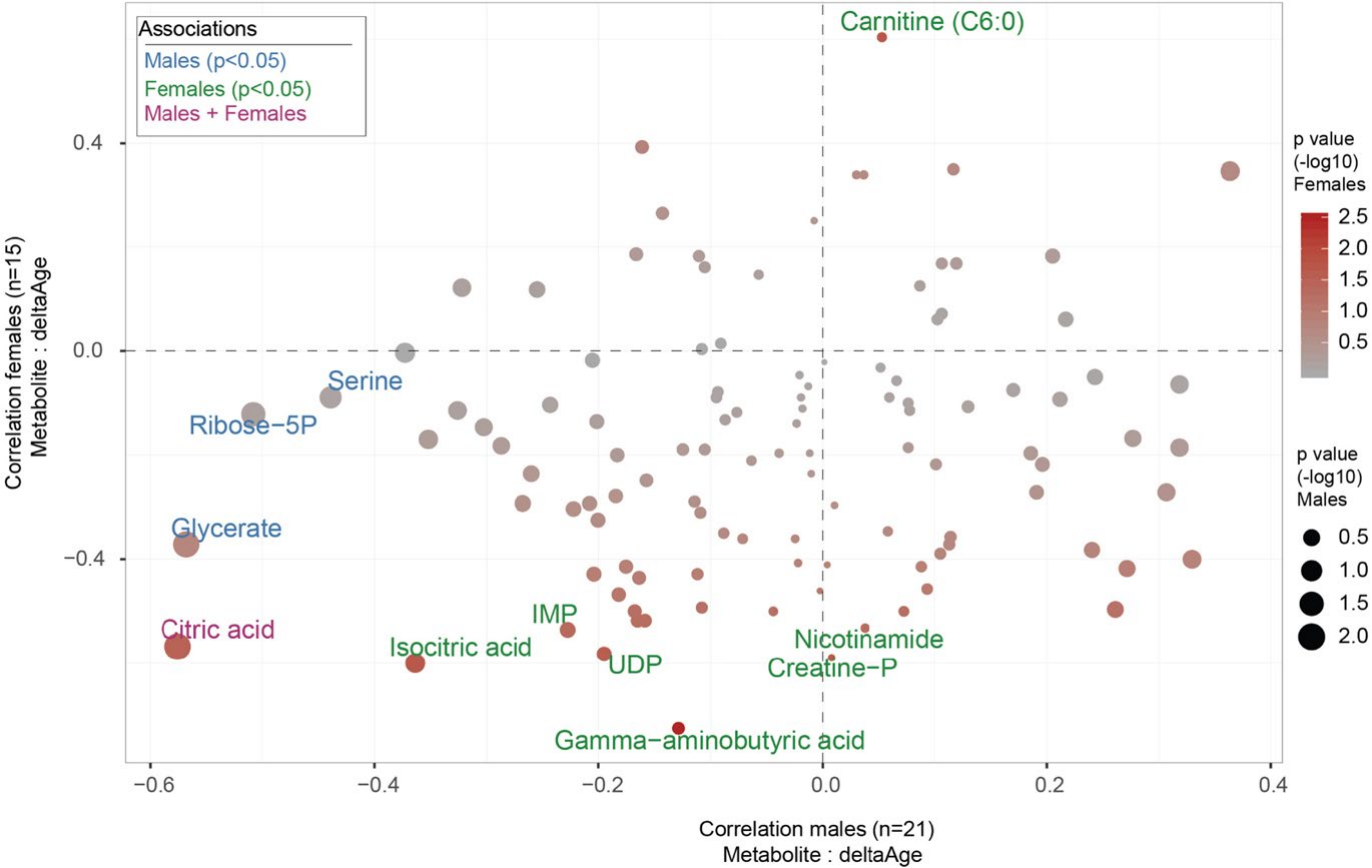
Female and male metabolic signatures of decelerated aging. Scatterplot comparing the metabolite correlation with deltaAge for either males (*x* axis, *n* = 21) or females (*y* axis, *n* = 15). Correlation considers only older adults. Related to the *x* axis Fig. 5D. Sizes represent –log10 *p* value of the correlation for males while color denotes –log10 *p* value of the correlation for females. Metabolite with text written in red is significant in both males and females, while in blue are those significant for males and in green are those significant for females (Pearson correlation, *p* < 0.05)

Finally, we asked ourselves how the metabolomics signatures of decelerated aging might be different between males and females in our cohort. To address this, we separated the cohort into two smaller populations (males, *n* = 21, and females, *n* = 15) and performed the same correlation of metabolites to deltaAge as generated previously (Fig. 5D on whole population, Fig. 7 in current analysis), focused on decelerated aging specifically. Here, we found interesting observations to emerge. Firstly, citric acid, the aforementioned component of the TCA cycle, was still significantly negatively associated with deltaAge in both males and females despite the smaller sample sizes. Secondly, nicotinamide, which may be sought after to boost NAD^+^ levels, was negatively associated with deltaAge in females, but not males. While the sample sizes here were low due to the separation of individuals into sub-cohorts of males and females, these findings nonetheless serve to both highlight again the strong relationship of the TCA cycle to age deceleration and suggest that disparities may exist between males and females when it comes to metabolic factors associated with decelerated aging.

## Discussion

We developed a physiological aging score, termed PhysiAge, based on systolic blood pressure, blood glucose, average steps per day, gender, and a normalization factor based on calendar age. Arguably, these parameters are highly accessible to individuals and open greater possibility for individuals to assess their own aging rates. Furthermore, our aging score allows for insights for how a person can directly modify their aging rate with suggestions to modify activity level or diet. Applying our model to a highly characterized and deeply phenotyped cohort allowed us to validate our model’s predictions beyond the association we found to mortality, and demonstrate that PhysiAge is more strongly associated with a decline in NAD^+^ levels, increase in oxidative stress markers, and decline in physiological functioning, compared to age alone. Encouraged by these results, we performed mass spec metabolomics on plasma samples from the same cohort, which led us to identify a metabolic signature of decelerated aging.

Several considerations accompany our study. Firstly, systolic blood pressure and blood glucose have influence on our model in a linear manner. While this was a choice in designing our model to offer simple interpretation, it suffers in that pathologically low levels of these parameters, i.e., individuals with hypoglycemia and hypotension, will possess physiAge scores skewed towards youthfulness. Since these are chronic conditions with potentially increased mortality rates [27, 28], the physiAge score would not be the most relevant metric to use for these individuals. Additionally, both systolic blood pressure and blood glucose plateau at older ages (apparent in Fig. 1A), resulting in the normalization factor used to generate PhysiAge plateauing as well (apparent in Fig. 2B). This implies that the normalization factor in PhysiAge becomes less important at older ages, putting more weight onto the parameters themselves (e.g., blood glucose and systolic blood pressure) for the final PhysiAge score. Therefore, the difference between an 80-year-old and an 82-year-old for their final PhysiAge score will be more heavily weighted by their actual physiology (e.g., blood glucose and systolic blood pressure) rather than age. This likely contributes to PhysiAge’s inherently strong association with mortality (Fig. 2E), since parameters in PhysiAge including blood glucose and systolic blood pressure are also health risk factors in general.

A second consideration for our work is that future versions of the PhysiAge model may benefit from including the non-linearly associated aging parameters we omitted. While this would convolute the interpretation of PhysiAge, it may offer additional resolution on the biological aging process and allow greater associations to mortality. A third consideration of our work is that our use of an average daily step count may not fully capture the broad range of activities that benefit physiological age, such as swimming and cycling. To resolve this, users may convert minutes of these particular activities into their equivalent step counts before calculating their PhysiAge [29]. Similarly, users who assess their step count via their mobile phone may have under-evaluated their true step count [30]. It is therefore advisable to use a reliable wearable device to assess step count for our model. A fourth consideration of our work, also regarding step counts, is that while immediately changing a person’s average daily step count will immediately change their PhysiAge score, the actual physiological effects may take longer to take effect biologically. Finally, a fifth consideration of our work is that our model uses age as a parameter in the prediction. While this approach has been used by us and others previously [7, 8, 31], it nonetheless results in a limit for how much age deceleration can occur. For example, an 80-year-old, even with the most youthful (though realistic) blood pressure, blood glucose, and steps per day, will never have the physiological age of a 20-year-old by our model. While a limitation, this may in fact provide a realistic view on how much one can modify their actual physiological age. Nonetheless, despite these limitations, our model provides a window into how choices influence physiological aging rates, and through this can provide motivation for healthier lifestyles.

Our work implicates the TCA cycle in human healthy aging. While discrepancies in the field exist [32], a vast body of literature also implicates abundance of TCA cycle metabolites in longevity, in line with our findings. For example, supplementation of the TCA cycle components malate and fumarate activate nuclear translocation of the FOXO/DAF-16 longevity gene, increase oxidative stress resistance, and extend lifespan in *C. elegans* worms [33]. Supplementation of TCA cycle component oxaloacetate also extends lifespan in worms dependent on the longevity gene FOXO/DAF-16 [34]. Supplementation of succinate, citrate, and alpha-ketoglutarate also extend lifespan in worms [35]. Furthermore, supplementation of citrate reduces energy status and extends lifespan in *Drosophila melanogaster* flies, and in mice fed a high-fat diet citrate improves metabolic health and memory [36]. Adding to this, TCA cycle genes are upregulated in Ames dwarf mice and little mice, which are both long-lived strains [37]. Functionality of the TCA cycle is also preserved when comparing long-lived to short-lived strains of Brown-Norway rats [38]. Remarkably, the TCA cycle intermediate alpha-ketoglutarate alone fed to mice extends lifespan and compresses morbidity [39]. In humans, a retrospective study using DNA methylation clocks to assess biological age found that supplementing alpha-ketoglutarate and certain other vitamins conferred an average of 8 years reduced biological age after an average of 7 months of use [40].

The finding that the TCA cycle was so strongly associated to decelerated aging in our data opens more questions. Firstly, it may be possible that beta-oxidation is feeding the TCA cycle. This is in line with observations that higher beta-oxidation is also present in long-lived mice [41]. Interestingly, our previous work in aging mice has suggested a shift in fat metabolism with aging [42]. However, dedicated metabolic flux experiments would be required to address this, which is difficult to perform in humans. Secondly, it may be that our measures on the TCA cycle in blood plasma serve as an indirect readout of mitochondrial activity in tissues. Indeed, our previous work with the same cohort used in PhysiAge validations implicated increased mitochondrial mass in the trained older adults [16, 25]. Again here, dedicated flux experiments would be required to trace whether muscle TCA cycle metabolism contributes to blood plasma TCA cycle components. Of additional consideration, it may be possible that the nature of metabolomics, being targeted, has had our investigational lens focus on known pathways including the TCA cycle. It would be of interest to perform these analyses using untargeted metabolomics. Of final note, our own study implicating the TCA cycle involved trained older adults, and therefore increasing TCA cycle constituents may in the end be best achieved simply by training more, rather than by supplementation strategies. In conclusion, these and our own findings support two main conclusions: (1) the function of the TCA cycle is causally linked to healthy aging across species, also in humans; and (2) an aging score derived from physiological parameters can serve as a proxy for individuals to assess their own biological aging.

## Methods

### Physiological parameters used for exploration and model building

The 2005–2006 data was accessed from the NHANES portal at CDC.gov. Participant age and gender was obtained from the DEMO_D.XPT file. BMI was obtained from the BMX_D.XPT data file. Heart rate and systolic and diastolic blood pressure were obtained from the BPX_D.XPT data file. Blood glucose was obtained from the BIOPRO_D.XPT data file. Step counts: To obtain average daily step counts, the paxraw_d.xpt accelerometer data file was used. Each of the 7 days of step data present per participant in the PAXSTEP data column was summed to get a maximum stem count per day and the 7 days were averaged to obtain an average daily step count per participant. Multiple individuals appeared to possess average daily step counts that were outliers in the population (e.g., their average daily step count was of several millions) and an outlier cutoff was applied, mainly for data visualization purposes, where individuals were removed if their average daily step count was defined as an outlier in a box-and-whisker plot [43] when considering all average daily step counts of the population.

### Calculation of phenotypic age

The 2005–2006 data was accessed from the NHANES portal at CDC.gov. Albumin, creatinine, blood glucose, and alkaline phosphatase values were obtained from the BIOPRO_D.XPT data file. CRP levels were obtained from the CRP_D.XPT data file. Lymphocyte, MCV values, red cell dist width, and WBCs were obtained from the CBC_D.XPT file. Participant age was obtained from the DEMO_D.XPT file. For each participant, phenotypic age was calculated based on calendar age and blood parameters according to the weights and equation developed by Levine and colleagues [7].

### PhysiAge data preparation

One datafile was compiled which contained participants which had data entries for all parameters explored in this study, including age, glucose, gender, BMI, systolic blood pressure, diastolic blood pressure, heart rate, phenotypic age, and average step count, for visualization purposes (Fig. 1). Any participant with a zero entry in one of those data columns was excluded from the analysis. For downstream validation, mortality information per participant was accessed at NHANES_2005_2006_MORT_2015_PUBLIC.dat from the CDC website. Data was filtered to only include remaining participants who possessed no NA entries in any of the parameters, and whose age was between and including 20 to 84. The 85 age entry was a code for 85 + in the NHANES dataset and included any individual aged 85 and older. Since precise ages were not available, this age group was not included in model building. These steps resulted in 3342 participants of ages distributed across a range from 20 to 84 years. Data was split into 80% training data and 20% testing data, equally across both sexes. Randomization seed was set to “1” for splitting the data using the set.seed function in R (version 3.5.1 [44]).

### PhysiAge model

A multiple linear model was made in R (version 3.5.1 [44] ) using the lm() function, to predict phenotypic age using gender, blood glucose, average steps per day, and systolic blood pressure as input parameters. The median predicted value per (calendar) age in the training data was used as a normalization parameter, specific for each year of age. The prediction from the model, with normalization, is termed PhysiAge. The final PhysiAge model can be defined as follows:

$$\mathrm{PhysiAge}={\left(\left(-18.5+\left({1.972}^{*}\mathrm{Sex}\right)+\left({3.348}^{*}\mathrm{Gluc}\right)+\left(-{0.0004715}^{*}\mathrm{Steps}\right)+\left({0.3988}^{*}\mathrm{SysBP}\right)\right)/{\mathrm{NF}}_{\mathrm{Age}}\right)}^{*} \mathrm{Age}$$

where “Sex” is a value of 1 for men and 0 for women, “Gluc” is blood glucose (mmol/L), “Steps” is average daily step count, SysBP is systolic blood pressure (mmHg), “NF_Age_” is a normalization factor specific for the calendar age of the individual, and “Age” is the calendar age in years. NF_Age_ for each calendar age is available in Supplemental Table 1.

### PhysiAge model variants

Several variants of the PhysiAge model were generated in this study. (1) Variants were produced identically as described above (section: *PhysiAge model*) but omitting once each parameter of the model, producing four additional models (omitting either Sex, Gluc, Steps, or SysBP). (2) All five parameters were used (Sex, Gluc, Steps, SysBP, Age) though different random data partitions were generated to make the 80% training and 20% testing datasets. Specifically, the function set.seed() was used in R version 3.5.1 [44], where randomization seed was set to either “1” (used in the actual final PhysiAge model) or 2–10 (used in assessing outcomes of 9 additional models with other random data partitions).

### PhysiAge aging insights

To demonstrate aging insights, four hypothetical individuals were created with blood pressures, blood glucose levels, and average daily step counts roughly representing the norm for their age. Either average daily step counts were varied (ranging from 5000 to 20,000 and increasing by 1000 unit increments) or blood glucose levels were varying (ranging from 4 to 10 and increasing by 1 unit increments). The hypothetical individuals then had their biological ages and deltaAges calculated for each varying value. DeltaAges were compared to how average daily step counts or blood glucose levels varied to demonstrate how each parameter influences the predicted age.

### MitoHealth cohort and participant parameters

The MitoHealth cohort is registered on clinicaltrials.gov (identifier NCT03666013). Participants were recruited in the community of Maastricht (The Netherlands) and its surroundings through advertisements at Maastricht University, in local newspapers, supermarkets, and at sports clubs. The study protocol was approved by the institutional Medical Ethical Committee and conducted in agreement with the declaration of Helsinki. All participants provided their written informed consent. Fifty-nine individuals possessed complete data on the parameters necessary to calculate PhysiAge. Data for either muscle metabolomics (NAD + and OPA) or physiological parameters (RAND 36, NOGD, and parameters for PhysiAge calculation) were accessed from Janssens et al. [25] or Grevendonck et al. [16] studies, respectively.

### Plasma collection

At 9 AM, after an overnight fast from 10 PM the preceding evening, blood plasma was collected. Samples were immediately stored at − 80 °C until further analysis.

### Metabolomics

Metabolomics was performed as previously described, with minor adjustments [45]. In a 2-mL tube, the following amounts of internal standard dissolved in water were added to each sample of approximately plasma: adenosine-^15^N_5_-monophosphate (5 nmol), adenosine-^15^N_5_-triphosphate (5 nmol), D_4_-alanine (0.5 nmol), D_7_-arginine (0.5 nmol), D_3_-aspartic acid (0.5 nmol), D_3_-carnitine (0.5 nmol), D_4_-citric acid (0.5 nmol), ^13^C_1_-citrulline (0.5 nmol), ^13^C_6_-fructose-1,6-diphosphate (1 nmol), guanosine-^15^N_5_-monophosphate (5 nmol), guanosine-^15^N_5_-triphosphate (5 nmol), ^13^C_6_-glucose (10 nmol), ^13^C_6_-glucose-6-phosphate (1 nmol), D_3_-glutamic acid (0.5 nmol), D_5_-glutamine (0.5 nmol), D_5_-glutathione (1 nmol), ^13^C_6_-isoleucine (0.5 nmol), D_3_-lactic acid (1 nmol), D_3_-leucine (0.5 nmol), D_4_-lysine (0.5 nmol), D_3_-methionine (0.5 nmol), D_6_-ornithine (0.5 nmol), D_5_-phenylalanine (0.5 nmol), D_7_-proline (0.5 nmol), ^13^C_3_-pyruvate (0.5 nmol), D_3_-serine (0.5 nmol), D_6_-succinic acid (0.5 nmol), D5-tryptophan (0.5 nmol), D_4_-tyrosine (0.5 nmol), D_8_-valine (0.5 nmol). After adding the internal standard mix, a 5-mm stainless-steel bead and polar phase solvents (for a total of 500 µL water and 500 µL MeOH) were added and samples were homogenized using a TissueLyser II (Qiagen, Hilden, Germany) for 5 min at a frequency of 30 times/s. Chloroform was added for a total of 1 mL to each sample before thorough mixing. Samples were then centrifuged for 10 min at 18,000* g*. The top layer, containing the polar phase, was transferred to a new 1.5-mL tube and dried using a vacuum concentrator at 60 °C. Dried samples were reconstituted in 100 µL 3:2 (v/v) methanol:water. Metabolites were analyzed using a Waters Acquity ultra-high-performance liquid chromatography system coupled to a Bruker Impact II™ Ultra-High Resolution Qq-Time-Of-Flight mass spectrometer. Samples were kept at 12 °C during analysis and 5 µL of each sample was injected. Chromatographic separation was achieved using a Merck Millipore SeQuant ZIC-cHILIC column (PEEK 100 × 2.1 mm, 3 µm particle size). Column temperature was held at 30 °C. Mobile phase consisted of (A) 1:9 (v/v) acetonitrile:water and (B) 9:1 (v/v) acetonitrile:water, both containing 5 mmol/L ammonium acetate. Using a flow rate of 0.25 mL/min, the LC gradient consisted of 100% B for 0–2 min, reach 0% B at 28 min, 0% B for 28–30 min, reach 100% B at 31 min, and 100% B for 31–32 min. Column re-equilibration is achieved at a flow rate of 0.4 mL/min at 100% B for 32–35 min. MS data were acquired using negative and positive ionization in full scan mode over the range of m/z 50–1200. Data were analyzed using Bruker TASQ software version 2.1.22.3. All reported metabolite intensities were normalized to dry tissue weight, as well as to internal standards with comparable retention times and response in the MS. Metabolite identification has been based on a combination of accurate mass, (relative) retention times, and fragmentation spectra, compared to the analysis of a library of standards (Sigma-Aldrich MSMLS). General repeatability of metabolite analysis was assessed for each metabolite using repeated measurements of a pooled sample. Additionally, all peak integrations were manually checked for quality in each sample, as large natural variance may skew pooled sample results.

### Statistical analyses and data visualization

Data was processed and analyses were performed with R version 3.5.1 [44] and Bioconductor version 3.7 [46]. Data was processed in part with the R package dplyr version 1.0.2 [47]. Correlations and statistical evaluations were performed in R using Spearman’s test. Comparisons of groups were performed in R using the Kruskal-Wallis test. Cross-correlations of parameters was performed in R using PerformanceAnalytics version 2.0.4. Visualization of data was performed using ggplot2 version 3.2.1 [48], ggpubr v 0.2.5 [49], ggrepel version 0.8.1 [50], with (certain) colors from RColorBrewer version 1.1–2 [51].

### Supplementary Information

Below is the link to the electronic supplementary material.

Supplementary file1 (CSV 1 KB)

Supplementary file2 (CSV 74 KB)

## Data Availability

Metabolomics data are available as supplementary materials accompanying this manuscript as processed abundance values. All other data supporting the findings of this study are either also available as supplementary materials accompanying this manuscript or are available from the corresponding author upon reasonable request.

## References

[1] López-Otín C, Blasco MA, Partridge L, Serrano M, Kroemer G (2013). The hallmarks of aging. Cell.

[2] de Magalhães JP, Stevens M, Thornton D (2017). The business of anti-aging science. Trends Biotechnol.

[3] Jylhävä J, Pedersen NL, Hägg S (2017). Biological age predictors. EBioMedicine.

[4] Galkin F (2020). Biohorology and biomarkers of aging: current state-of-the-art, challenges and opportunities. Ageing Res Rev.

[5] Zhavoronkov A, Mamoshina P (2019). Deep aging clocks: the emergence of AI-based biomarkers of aging and longevity. Trends Pharmacol Sci.

[6] Robinson O, Lau CHE (2020). Measuring biological age using metabolomics. Aging.

[7] Levine ME (2018). An epigenetic biomarker of aging for lifespan and healthspan. Aging (Albany. NY).

[8] McIntyre RL, Rahman M, Vanapalli SA, Houtkooper RH, Janssens GE (2021). Biological age prediction from wearable device movement data identifies nutritional and pharmacological interventions for healthy aging. Front Aging.

[9] Kim Y (2022). Higher diet quality relates to decelerated epigenetic aging. Am J Clin Nutr.

[10] Crous-Bou M (2014). Mediterranean diet and telomere length in Nurses’ Health study: Population based cohort study. BMJ.

[11] Okazaki S (2020). Decelerated epigenetic aging associated with mood stabilizers in the blood of patients with bipolar disorder. Transl Psychiatry.

[12] Castillo-Quan JI (2016). Lithium promotes longevity through GSK3/NRF2-dependent hormesis. Cell Rep.

[13] Nespital T, Neuhaus B, Mesaros A, Pahl A, Partridge L (2021). Lithium can mildly increase health during ageing but not lifespan in mice. Aging Cell.

[14] Evason K, Collins JJ, Huang C, Hughes S, Kornfeld K (2008). Valproic acid extends Caenorhabditis elegans lifespan. Aging Cell.

[15] Houtkooper RH, Williams RW, Auwerx J (2010). Metabolic networks of longevity. Cell.

[16] Grevendonk L (2021). Impact of aging and exercise on skeletal muscle mitochondrial capacity, energy metabolism, and physical function. Nat Commun.

[17] Srikanthan P, Karlamangla AS (2014). Muscle mass index as a predictor of longevity in older adults. Am J Med.

[18] Basu R (2003). Mechanisms of the age-associated deterioration in glucose tolerance: contribution of alterations in insulin secretion, action, and clearance. Diabetes.

[19] Santos MAA (2013). Does the aging process significantly modify the mean heart rate?. Arq Bras Cardiol.

[20] Singh JN, Nguyen T, Kerndt CC, Dhamoon AS. Physiology, blood pressure age related changes. In: StatPearls [Internet]. Treasure Island (FL): StatPearls Publishing; 2023. 30725982

[21] Yamamoto N (2018). Daily step count and all-cause mortality in a sample of Japanese elderly people: a cohort study. BMC Public Health.

[22] Abell JG (2018). Association between systolic blood pressure and dementia in theWhitehall II cohort study: role of age, duration, and threshold used to define hypertension. Eur Heart J.

[23] Flint AC (2019). Effect of systolic and diastolic blood pressure on cardiovascular outcomes. N Engl J Med.

[24] Grundy SM, Brewer HB, Cleeman JI, Smith SC, Lenfant C (2004). Definition of metabolic syndrome. Arterioscler Thromb Vasc Biol.

[25] Janssens GE (2022). Healthy aging and muscle function are positively associated with NAD+ abundance in humans. Nat Aging.

[26] Zapata-Pérez R, Wanders RJA, Karnebeek CDM, Houtkooper RH (2021). NAD + homeostasis in human health and disease. EMBO Mol Med.

[27] McCoy RG (2012). Increased mortality of patients with diabetes reporting severe hypoglycemia. Diabetes Care.

[28] Masaki KH (1998). Orthostatic hypotension predicts mortality in elderly men: the Honolulu Heart Program. Circulation.

[29] Miller R, Brown W, Tudor-Locke C (2016). But what about swimming and cycling? How to “count” non-ambulatory activity when using pedometers to assess physical activity. J Phys Act Heal.

[30] Piccinini F, Martinelli G, Carbonaro A (2020). Accuracy of mobile applications versus wearable devices in long-term step measurements. Sensors (Switzerland).

[31] El Khoury LY (2019). Systematic underestimation of the epigenetic clock and age acceleration in older subjects. Genome Biol.

[32] Cheng S (2015). Distinct metabolomic signatures are associated with longevity in humans. Nat Commun.

[33] Edwards CB, Copes N, Brito AG, Canfield J, Bradshaw PC (2013). Malate and fumarate extend lifespan in Caenorhabditis elegans. PLoS ONE.

[34] Williams DS, Cash A, Hamadani L, Diemer T (2009). Oxaloacetate supplementation increases lifespan in caenorhabditis elegans through an AMPK/FOXO-dependent pathway. Aging Cell.

[35] Edwards C (2015). Mechanisms of amino acid-mediated lifespan extension in Caenorhabditis elegans. BMC Genet.

[36] Fan SZ (2021). Dietary citrate supplementation enhances longevity, metabolic health, and memory performance through promoting ketogenesis. Aging Cell.

[37] Amador-Noguez D, Yagi K, Venable S, Darlington G (2004). Gene expression profile of long-lived Ames dwarf mice and Little mice. Aging Cell.

[38] Perron JT, Tyson RL, Sutherland GR (2000). Maintenance of tricarboxylic acid cycle kinetics in Brown-Norway Fischer 344 rats may translate to longevity. Neurosci Lett.

[39] Asadi Shahmirzadi A (2020). Alpha-ketoglutarate, an endogenous metabolite, extends lifespan and compresses morbidity in aging mice. Cell Metab.

[40] Demidenko O (2021). Rejuvant®, a potential life-extending compound formulation with alpha-ketoglutarate and vitamins, conferred an average 8 year reduction in biological aging, after an average of 7 months of use, in the TruAge DNA methylation test. Aging (Albany. NY).

[41] Bartke A, Westbrook R (2012). Metabolic characteristics of long-lived mice. Front Genet.

[42] Houtkooper RH (2011). The metabolic footprint of aging in mice. Sci Rep.

[43] Tukey JW. Exploratory data analysis. Boston: Addison-Wesley; 1977.

[44] Team, \proglangR Development Core. \proglang{R}: a language and environment for statistical computing. R Foundation for Statistical Computing Vienna, Austria. ISBN 3–900051–07–0, URL http://ww. 2010.

[45] Molenaars M (2020). A conserved mito-cytosolic translational balance links two longevity pathways. Cell Metab.

[46] Gentleman RC, Carey VJ, Bates DM, Bolstad B, Dettling M, Dudoit S, et al. Bioconductor: open software development for computational biology and bioinformatics. Genome Biol. 2004;5(10):R80. 10.1186/gb-2004-5-10-r80. 10.1186/gb-2004-5-10-r80 PMC545600 15461798

[47] Wickham H, François R, Henry L, Müller K, Vaughan D. dplyr: a grammar of data manipulation. 2023. https://dplyr.tidyverse.org, https://github.com/tidyverse/dplyr.

[48] Wickham H (2011). Ggplot2. Wiley Interdiscip Rev Comput Stat.

[49] Kassambara A. ggpubr: ‘ggplot2’ based publication ready plots. R package version 0.2.5. 2020. https://CRAN.R-project.org/package=ggpubr.

[50] Slowikowski K. ggrepel: automatically position non-overlapping text labels with ‘ggplot2’. R package version 0.8.1. 2019. https://CRAN.R-project.org/package=ggrepel.

[51] Neuwirth E. RColorBrewer: ColorBrewer palettes. R package version 1.1-2. 2014. https://CRAN.R-project.org/package=RColorBrewer.

